# Crystal structures of two chiral piperidine derivatives: 1-[(1*R*)-2-hy­droxy-1-phenyl­eth­yl]piperidin-4-one and 8-[(1*S*)-1-phenyl­eth­yl]-1,4-dioxa-8-aza­spiro­[4.5]decane-7-thione

**DOI:** 10.1107/S2056989015017119

**Published:** 2015-09-26

**Authors:** Nancy Romero, Sylvain Bernès, Luis F. Roa, Joel L. Terán, Dino Gnecco

**Affiliations:** a Universidad Juárez Autónoma de Tabasco, División Académica de Ciencias Básicas, Km. 1 carretera Cunduacán, Jalpa de Méndez AP 24, Cunduacán, Tabasco, Mexico; b Instituto de Física, Benemérita Universidad Autónoma de Puebla, Av. San Claudio y 18 Sur, 72570 Puebla, Pue., Mexico; c Centro de Química, Instituto de Ciencias, Benemérita Universidad Autónoma de Puebla, 72570 Puebla, Pue., Mexico

**Keywords:** crystal structure, piperidine, piperidone, thione, ring conformation

## Abstract

The crystal structures of the two title piperidine derivatives show different conformations for the six-membered heterocycle. The *N*-substituted 4-piperidinone 1-[(1*R*)-2-hy­droxy-1-phenyl­eth­yl]piperidin-4-one, C_13_H_17_NO_2_, (I), has a chair conformation, while the piperidine substituted in position 2 with a thio­carbonyl group, 8-[(1*S*)-1-phenyl­eth­yl]-1,4-dioxa-8-aza­spiro­[4.5]decane-7-thione, C_15_H_19_NO_2_S, (II), features a half-chair conformation. Comparison of the two structures, and data retrieved from the literature, suggests that the conformational flexibility is mainly related to the hybridization state of the C atom α to the piperidinic N atom: a C*sp* ^3^ atom favours the chair conformer, while a C*sp* ^2^ atom distorts the ring towards a half-chair conformer. In the crystal structure of (I), weak C—H⋯O hydrogen bonds link the mol­ecules into supra­molecular chains propagating along the *b*-axis direction. In the crystal of (II), the mol­ecules are linked by weak C—H⋯S contacts into supra­molecular chains propagating along the *b*-axis direction.

## Abstract 2

The conformation of the piperidine ring is modified by the hybridization state of the C atom in the α-position to the piperidinic N atom.

## Chemical context   

The 4-piperidone scaffold has been used as a building block for the synthesis of more complex heterocyclic compounds. An example is the one-pot three-step synthesis of fentanyl [*N*-(1-phenethyl-4-piperid­yl) propionanilide], a strong agonist of μ-opioid receptors, used for its potent analgesic activity. This industrial synthesis, patented by Janssen Pharmaceutica (Gupta *et al.*, 2010 ▸) employs 4-piperidone hydro­chloride monohydrate as the starting material. The range of biological activity for 4-piperidone derivatives is quite broad, including anti-inflammatory, anti­cancer, anti­bacterial and anti­fungal properties. For this reason, new synthetic methods are being sought proactively in this field (*e.g*. Tortolani & Poss, 1999 ▸; Davis *et al.*, 2001 ▸; Das *et al.*, 2010 ▸). For our part, our emphasis is on the synthesis of chiral *N*-substituted piperidone derivatives (*e.g*. Romero *et al.*, 2007 ▸).
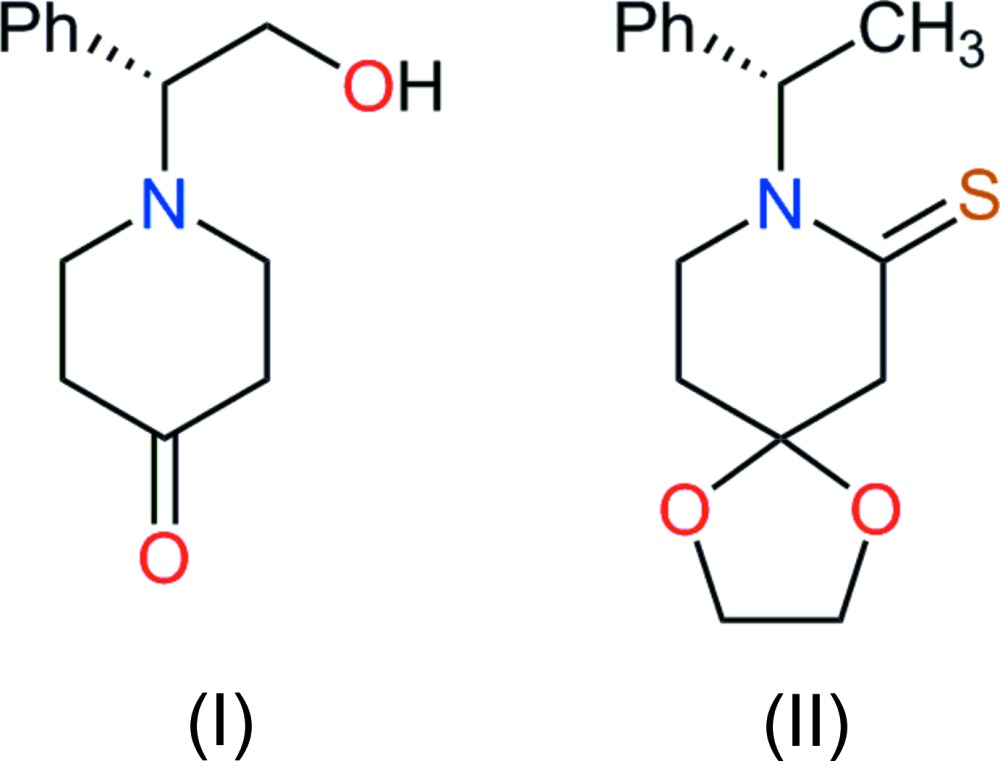


In this context, X-ray crystallography is a potent tool to assess the conformational modifications experienced by the piperidine heterocycle while its substitution pattern is altered along a synthetic route. The pair of structures reported here illustrates such conformational flexibility in this chemistry.

## Structural commentary   

**Figure 1:**
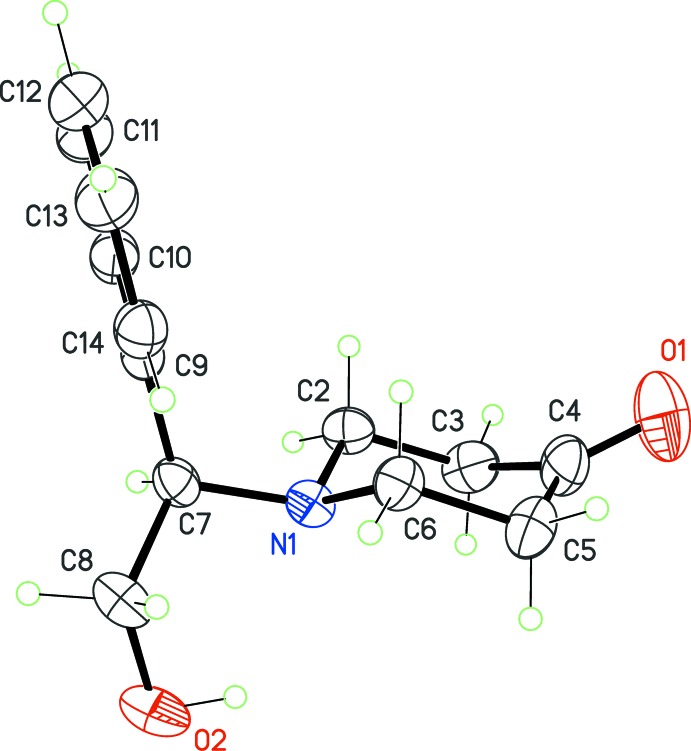
The mol­ecular structure of (I), with displacement ellipsoids for non-H atoms at the 30% probability level.

The first piperidin-4-one derivative [(I), Fig. 1 ▸] is a non-sterically hindered mol­ecule, and thus adopts the most stable chair conformation for the six-membered heterocycle. The total puckering amplitude is *Q* = 0.553 (3) Å, and the Cremer parameters are θ = 168.8 (3)° and φ = 171.8 (18)°. The deviation from the ideal conformation, θ = 180°, may be related to the heterocyclic nature of the ring, with short C—N bond lengths and longer C—C bond lengths, as expected. Moreover, atom C4 has a geometry consistent with its *sp* ^2^ hybridization state, while N1 is essentially tetra­hedral, with the lone pair occupying the axial position. The equatorial group substituting this N atom is rigid, as a result of its chiral character. However, the spatial orientation of this group allows the hydroxyl group to inter­act with the nitro­gen lone pair, stabilizing the observed mol­ecular conformation.

The chair conformation for the piperidone in (I) was previously observed in related compounds based on the same heterocycle (Vijayakumar *et al.*, 2010 ▸; Rajesh *et al.*, 2010*a* ▸, 2012 ▸). Apparently, the only significant variation allowed for this system is for the N atom, which may approach a planar–trigonal geometry (Shahani *et al.*, 2010 ▸; Rajesh *et al.*, 2010*b* ▸).

**Figure 2:**
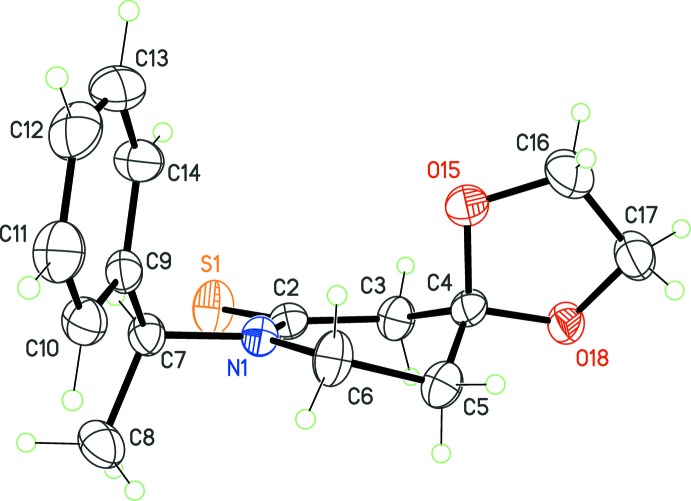
The mol­ecular structure of (II), with displacement ellipsoids for non-H atoms at the 30% probability level.

The chair conformation of (I) is, however, different from that observed for (II), derived from piperidine-2-thione (Fig. 2 ▸). In that case, the half-chair form is found in the crystal structure, characterized by a puckering amplitude *Q* = 0.513 (3) Å, and Cremer parameters θ = 127.5 (3)° and φ = 29.29 (5)° (ideal values: θ = 129.2° and φ = 30°). The N atom has a planar environment, the sum of angles about this center being 360°. This conformer is identical to one of the stable forms reported for piperidin-2-one (known as δ-valerolatcam): microwave spectroscopy indicated that for δ-valerolatcam, two conformers are stabilized in the gas phase, the half-chair form and the twist form (Kuze *et al.*, 1999 ▸). δ-Valerolatcam is actually comparable to (II), because in both mol­ecules C4 has the same *sp* ^3^ hybridization. In (II), the spiro atom C4 is part of the 1,3-dioxolane ring. The slightly twisted half-chair conformation for this ring is common. The two rings are almost perpendicular, as reflected in the dihedral angle between their mean-planes of 76.4 (2)°.

## Effect of hybridization on ring conformation   

Since the ring conformation in (II) seems not to be related to any intra­molecular strong inter­action nor the hybridization modification from *sp* ^2^ to *sp* ^3^ at C4, it should be a consequence of the presence of the thio­carbonyl functionality at C2. This center is in a state very close to pure *sp* ^2^ hybridization. This is reflected in the bond length for the C=S group, 1.677 (3) Å, close to the mean value of 1.669 Å computed from almost 10000 thio­carbonyl bonds retrieved from the organics subset of the CSD (Version 5.36 with all updates; Groom & Allen, 2014 ▸. The restriction to *sp* ^2^-C centers is applied by requiring the C atom to be linked to exactly three atoms and the S atom to be linked to exactly one atom). Indeed, long C=S bonds, above 1.75 Å, are found in compounds including mol­ecules having a propensity to form hydrogen bonds, like thio­urea (Weber, 1984 ▸), thio­urea derivatives (Busschaert *et al.*, 2011 ▸; Chumakov *et al.*, 2006 ▸), and tri­thio­carbonic acid (Krebs & Gattow, 1965 ▸), among others. In the case of a single C—S bond based on a *sp* ^3^-hybridized C atom, the bond length is sharply distributed around 1.81 Å.

The other factor contributing to the ring conformation in (II) is the absence of the hydroxyl group in the chiral moiety, making the heterocyclic N atom inert towards potential inter­actions. The lone pair should thus be oriented randomly above and below the piperidine mean plane, through nitro­gen inversion, characterized by a low energy barrier in the gas and solution phases. Both features, the planar N atom and the neighboring *sp* ^2^-C atom, generate the half-chair conformation observed for the piperidine-2-thione core. In the present case, it is difficult to determine whether one feature dominates, or both are of importance for stabilizing the half-chair conformation. However, for the 25 hits corresponding to piperidine-2-ones deposited in the CSD, 21 of them present the same conformation as in (II), with C4 as the flap atom for the half-chair. In three cases, the puckering amplitude of the half-chair is close to 0 Å (Woydt *et al.*, 1991 ▸; Bolla *et al.*, 2014 ▸), and in one case, the ring presents a twist-boat conformation (Sanfilippo *et al.*, 1992 ▸). In contrast, piperidine derivatives are stabilized almost universally in the chair conformation, with very few exceptions in some disordered structures (Thirumaran *et al.*, 2009 ▸). These rules hold regardless of the substituent on the N atom. Applying these general rules to compounds (I) and (II), we thus infer that the ring conformation is mainly determined by the hybridization state of the C atom in position α to the piperidinic N atom.

## Supra­molecular features   

In the crystal of (I), weak C—H⋯O hydrogen bonds link the mol­ecules into supra­molecular chains propagating along the *b*-axis direction (Table 1 ▸).

The crystal structure of (II) is based on weak inter­molecular C—H⋯S contacts involving one methyl­ene group of the dioxolane ring and the thio­carbonyl functionality (Table 2 ▸), which forms chains along the 2_1_ symmetry axis parallel to [010].

## Synthesis and crystallization   

**Figure 3:**
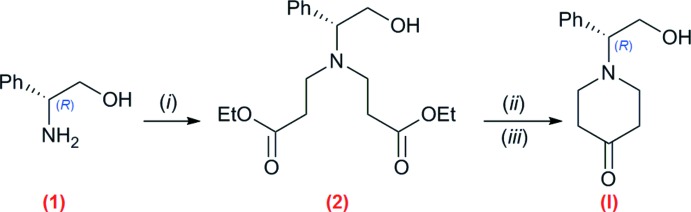
Synthesis of (I). Reaction conditions: (*i*) ethyl acrylate, MeOH, 298 K, 12 h; (*ii*) Na/MeOH, benzene, reflux, 5 h; (*iii*) AcOH/H_2_O (30% *v*/*v*), reflux.

**Compound (I)**. The synthesis is illustrated in Fig. 3 ▸. A solution of compound (1), (*R*)-(−)-2-phenyl­glycinol (5.65 g, 41.2 mmol) with an excess of ethyl acrylate in methanol (60 mL), was stirred overnight at 298 K. The reaction mixture was concentrated, and the crude purified by column chromatography (SiO_2_, CH_2_Cl_2_:MeOH, 97:3), to afford (2) as a colorless oil (98%). An amount of (2) (40.6 mmol) was added to a mixture of MeONa in anh. benzene. After refluxing the mixture for 5 h, a solid was obtained, which was filtered and dried in air. This solid was treated with AcOH:water (30%, *v*/*v*) until pH = 1, initiating the deca­rboxylation process. The mixture was refluxed until gas evolution stopped. After cooling down to 298 K, pH was adjusted to 7 with NaHCO_3_, and the mixture was washed with CH_2_Cl_2_ (3 × 50 ml). The organic phase was dried over Na_2_SO_4_, and concentrated. Compound (I) was purified by column chromatography (SiO_2_, CH_2_Cl_2_:MeOH, 95:5). Compound (I) was obtained in 80% yield, and was recrystallized from an AcOEt:*n*-hexane mixture (1:1).

**Figure 4:**
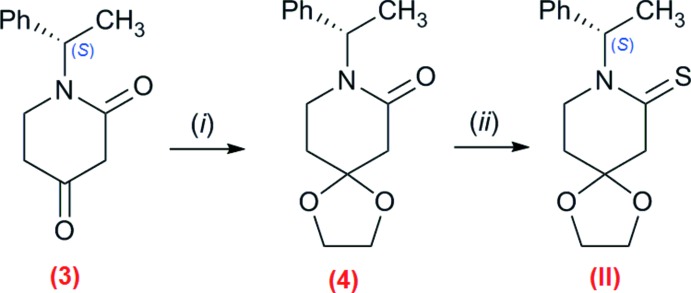
Synthesis of (II). Reaction conditions: (*i*) ethyl­ene glycol, *p*-TSA, anhydrous benzene, reflux, 4 h; (*ii*) Lawesson’s reagent in anhydrous toluene, 313 K, 1 h.

**Compound (II)**. The synthesis is illustrated in Fig. 4 ▸. The synthesis of compound (3), (*S*)-(−)-phenyl­ethyl­piperi-2,4-dione, has been reported previously (Romero *et al.*, 2013 ▸; see compound 5 in Fig. 1 ▸ of this report). To a solution of (3) in 50 mL of dry benzene, was added ethyl­ene glycol (0.2 mL, 3.4 mmol) and a catalytic amount of *p*-TSA. The mixture was refluxed until water formation, collected with a Dean–Stark trap, stopped. Then, the reaction mixture was cooled down to room temperature, treated with brine, and washed with CH_2_Cl_2_ (3 × 50 mL). The organic phase was dried over Na_2_SO_4_, and then concentrated under reduced pressure. The crude reaction was purified by column chromatography (SiO_2_, AcOEt:petroleum benzine), to afford compound (4) as a white oil, in 95% yield. Next, a suspension of Lawesson’s reagent (0.234 g, 0.578 mmol) in dry toluene (60 mL) was refluxed until complete dissolution of the reagent. The solution was cooled to 313 K, and a solution of (4) in anh. toluene (0.151 g, 0.578 mmol, 20 mL) was added (Romero *et al.*, 2007 ▸). The reaction mixture was stirred for 1 h to give (II) in 90% yield, after purification by column chromatography (SiO_2_, petroleum ether:dicloro­methane). The product was recrystallized from CH_2_Cl_2_:*n*-hexane (1:1).

## Refinement   

Crystal data, data collection and structure refinement details are summarized in Table 3 ▸. All C-bound H atoms were placed in calculated positions, and refined as riding on their carrier atoms, and with C—H bond lengths fixed at 0.93 (aromatic CH), 0.96 (methyl CH_3_), 0.97 (methyl­ene CH_2_), or 0.98 Å (methine CH). For (I), the hydroxyl H atom, H2, was first found in a difference map. Its position was fixed in the last least-squares cycles, with O2—H2 = 0.91 Å. For all H atoms, the isotropic displacement parameters were calculated as *U*_iso_(H) = *xU*_eq_(carrier atom), where *x* = 1.5 for methyl and hydroxyl H atoms, and *x* = 1.2 otherwise. The absolute configuration for chiral centers C7 in (I) and (II) was assumed from the chirality of starting materials used for the synthesis (see previous section). In the case of (II), which contains one site producing anomalous scattering, the expected enanti­omer was confirmed by the refinement of the Flack parameter (Parsons *et al.*, 2013 ▸).

## Supplementary Material

Crystal structure: contains datablock(s) I, II, global. DOI: 10.1107/S2056989015017119/xu5868sup1.cif

Structure factors: contains datablock(s) I. DOI: 10.1107/S2056989015017119/xu5868Isup2.hkl

Structure factors: contains datablock(s) II. DOI: 10.1107/S2056989015017119/xu5868IIsup3.hkl

Supporting information file. DOI: 10.1107/S2056989015017119/xu5868Isup4.cml

Supporting information file. DOI: 10.1107/S2056989015017119/xu5868IIsup5.cml

CCDC references: 1424094, 1424093

Additional supporting information:  crystallographic information; 3D view; checkCIF report

## Figures and Tables

**Table 1:** Hydrogen-bond geometry (Å, °) for (I)

*D*—H⋯*A*	*D*—H	H⋯*A*	*D*⋯*A*	*D*—H⋯*A*
C3—H3*A*⋯O1^i^	0.97	2.49	3.246 (4)	135

Symmetry code: (i) 
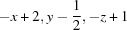
.

**Table 2:** Hydrogen-bond geometry (Å, °) for (II)

*D*—H⋯*A*	*D*—H	H⋯*A*	*D*⋯*A*	*D*—H⋯*A*
C16—H16*B*⋯S1^i^	0.97	2.85	3.709 (5)	148

Symmetry code: (i) 
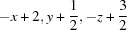
.

**Table 3:** Experimental details

	(I)	(II)
Crystal data		
Chemical formula	C_13_H_17_NO_2_	C_15_H_19_NO_2_S
*M*_r_	219.27	277.37
Crystal system, space group	Monoclinic, *P*2_1_	Orthorhombic, *P*2_1_2_1_2_1_
Temperature (K)	296	296
*a*, *b*, *c* (Å)	9.7590 (11), 6.8952 (10), 9.7980 (14)	5.9731 (13), 14.948 (3), 16.127 (3)
α, β, γ (°)	90, 114.348 (9), 90	90, 90, 90
*V* (Å^3^)	600.67 (15)	1439.9 (5)
*Z*	2	4
Radiation type	Mo *K*α	Mo *K*α
μ (mm^−1^)	0.08	0.22
Crystal size (mm)	0.60 × 0.17 × 0.12	0.60 × 0.38 × 0.36

Data collection		
Diffractometer	Bruker P4	Bruker P4 diffractometer
Absorption correction	–	ψ scan (*XSCANS*; Fait, 1996 ▸)
*T*_min_, *T*_max_	–	0.760, 0.922
No. of measured, independent and observed [*I* > 2σ(*I*)] reflections	2700, 1341, 1050	3886, 2631, 2007
*R*_int_	0.021	0.029
(sin θ/λ)_max_ (Å^−1^)	0.595	0.650

Refinement		
*R*[*F* ^2^ > 2σ(*F* ^2^)], *wR*(*F* ^2^), *S*	0.036, 0.083, 1.04	0.044, 0.120, 1.06
No. of reflections	1341	2631
No. of parameters	146	174
No. of restraints	1	0
H-atom treatment	H-atom parameters constrained	H-atom parameters constrained
Δρ_max_, Δρ_min_ (e Å^−3^)	0.11, −0.11	0.19, −0.24
Absolute structure	Assigned from the synthesis	Flack *x* determined using 483 quotients [(*I* ^+^)−(*I* ^−^)]/[(*I* ^+^)+(*I* ^−^)] (Parsons *et al.*, 2013 ▸)
Absolute structure parameter	–	0.08 (7)

Computer programs: *XSCANS* (Fait, 1996 ▸), *SHELXS97* and *SHELXTL* (Sheldrick, 2008 ▸) and *SHELXL2014* (Sheldrick, 2015 ▸).

## supplementary crystallographic information

### (I) 1-[(1R)-2-Hydroxy-1-phenylethyl]piperidin-4-one Crystal data

**Table:**

C_13_H_17_NO_2_	*F*(000) = 236
*M**_r_* = 219.27	*D*_x_ = 1.212 Mg m^−^^3^
Monoclinic, *P*2_1_	Mo *K*α radiation, λ = 0.71073 Å
*a* = 9.7590 (11) Å	Cell parameters from 53 reflections
*b* = 6.8952 (10) Å	θ = 3.7–11.1°
*c* = 9.7980 (14) Å	µ = 0.08 mm^−^^1^
β = 114.348 (9)°	*T* = 296 K
*V* = 600.67 (15) Å^3^	Plate, pale yellow
*Z* = 2	0.60 × 0.17 × 0.12 mm

### (I) 1-[(1R)-2-Hydroxy-1-phenylethyl]piperidin-4-one Data collection

**Table:**

Bruker P4 diffractometer	*R*_int_ = 0.021
Radiation source: fine-focus sealed tube	θ_max_ = 25.0°, θ_min_ = 2.3°
Graphite monochromator	*h* = −11→4
2θ/ω scans	*k* = −1→8
2700 measured reflections	*l* = −10→11
1341 independent reflections	3 standard reflections every 97 reflections
1050 reflections with *I* > 2σ(*I*)	intensity decay: 0.5%

### (I) 1-[(1R)-2-Hydroxy-1-phenylethyl]piperidin-4-one Refinement

**Table:**

Refinement on *F*^2^	Secondary atom site location: difference Fourier map
Least-squares matrix: full	Hydrogen site location: inferred from neighbouring sites
*R*[*F*^2^ > 2σ(*F*^2^)] = 0.036	H-atom parameters constrained
*wR*(*F*^2^) = 0.083	*w* = 1/[σ^2^(*F*_o_^2^) + (0.0372*P*)^2^ + 0.0246*P*] where *P* = (*F*_o_^2^ + 2*F*_c_^2^)/3
*S* = 1.04	(Δ/σ)_max_ < 0.001
1341 reflections	Δρ_max_ = 0.11 e Å^−^^3^
146 parameters	Δρ_min_ = −0.11 e Å^−^^3^
1 restraint	Extinction correction: *SHELXL2014* (Sheldrick, 2015), Fc^*^=kFc[1+0.001xFc^2^λ^3^/sin(2θ)]^-1/4^
0 constraints	Extinction coefficient: 0.040 (6)
Primary atom site location: structure-invariant direct methods	Absolute structure: Assigned from the synthesis

### (I) 1-[(1R)-2-Hydroxy-1-phenylethyl]piperidin-4-one Fractional atomic coordinates and isotropic or equivalent isotropic displacement parameters (Å^2^)

**Table:**

	*x*	*y*	*z*	*U*_iso_*/*U*_eq_	
N1	0.8276 (2)	0.3211 (3)	0.1078 (2)	0.0427 (5)	
O1	0.9225 (3)	0.7423 (5)	0.4197 (3)	0.1214 (11)	
O2	0.7952 (2)	−0.0767 (3)	0.0760 (3)	0.0797 (7)	
H2	0.8389	0.0052	0.1543	0.120*	
C2	0.9480 (3)	0.4619 (4)	0.1324 (3)	0.0502 (7)	
H2B	0.9068	0.5750	0.0700	0.060*	
H2C	1.0229	0.4047	0.1036	0.060*	
C3	1.0222 (3)	0.5237 (5)	0.2969 (3)	0.0629 (9)	
H3A	1.0773	0.4150	0.3575	0.075*	
H3B	1.0934	0.6273	0.3082	0.075*	
C4	0.9084 (4)	0.5914 (6)	0.3506 (3)	0.0708 (10)	
C5	0.7746 (3)	0.4646 (5)	0.3102 (3)	0.0613 (9)	
H5A	0.6983	0.5313	0.3318	0.074*	
H5B	0.8025	0.3476	0.3705	0.074*	
C6	0.7102 (3)	0.4101 (4)	0.1448 (2)	0.0491 (7)	
H6A	0.6277	0.3196	0.1226	0.059*	
H6B	0.6716	0.5252	0.0840	0.059*	
C7	0.7704 (3)	0.2371 (4)	−0.0447 (3)	0.0461 (7)	
H7A	0.8596	0.2074	−0.0631	0.055*	
C8	0.6978 (3)	0.0421 (4)	−0.0420 (3)	0.0626 (8)	
H8A	0.6054	0.0634	−0.0296	0.075*	
H8B	0.6726	−0.0237	−0.1368	0.075*	
C9	0.6741 (3)	0.3692 (4)	−0.1714 (2)	0.0460 (7)	
C10	0.7397 (3)	0.4820 (5)	−0.2455 (3)	0.0570 (8)	
H10A	0.8428	0.4733	−0.2180	0.068*	
C11	0.6544 (4)	0.6079 (6)	−0.3602 (3)	0.0737 (9)	
H11A	0.7010	0.6837	−0.4072	0.088*	
C12	0.5013 (4)	0.6203 (6)	−0.4042 (3)	0.0786 (10)	
H12A	0.4440	0.7028	−0.4819	0.094*	
C13	0.4338 (4)	0.5101 (5)	−0.3326 (3)	0.0684 (9)	
H13A	0.3304	0.5181	−0.3620	0.082*	
C14	0.5188 (3)	0.3872 (4)	−0.2169 (3)	0.0541 (7)	
H14A	0.4716	0.3151	−0.1684	0.065*	

### (I) 1-[(1R)-2-Hydroxy-1-phenylethyl]piperidin-4-one Atomic displacement parameters (Å^2^)

**Table:**

	*U*^11^	*U*^22^	*U*^33^	*U*^12^	*U*^13^	*U*^23^
N1	0.0361 (11)	0.0413 (12)	0.0519 (12)	0.0012 (11)	0.0192 (9)	0.0002 (11)
O1	0.130 (2)	0.119 (2)	0.104 (2)	−0.022 (2)	0.0365 (16)	−0.062 (2)
O2	0.0924 (15)	0.0412 (12)	0.1027 (14)	0.0085 (13)	0.0374 (12)	0.0124 (13)
C2	0.0445 (14)	0.0492 (17)	0.0558 (14)	−0.0064 (15)	0.0194 (11)	0.0027 (14)
C3	0.0533 (16)	0.068 (2)	0.0566 (15)	−0.0138 (18)	0.0114 (13)	0.0026 (17)
C4	0.080 (2)	0.077 (3)	0.0418 (14)	0.003 (2)	0.0114 (15)	−0.0097 (18)
C5	0.0580 (16)	0.080 (2)	0.0481 (14)	0.0064 (19)	0.0243 (13)	−0.0025 (17)
C6	0.0442 (13)	0.0563 (18)	0.0487 (13)	0.0012 (16)	0.0210 (11)	−0.0009 (15)
C7	0.0480 (14)	0.0403 (14)	0.0555 (15)	0.0030 (14)	0.0270 (12)	−0.0042 (14)
C8	0.0660 (19)	0.0403 (16)	0.0795 (19)	−0.0022 (16)	0.0281 (16)	−0.0061 (16)
C9	0.0559 (15)	0.0404 (17)	0.0435 (13)	−0.0022 (15)	0.0222 (12)	−0.0068 (14)
C10	0.0677 (17)	0.0555 (18)	0.0520 (15)	−0.0106 (17)	0.0287 (14)	−0.0061 (16)
C11	0.104 (3)	0.061 (2)	0.0584 (17)	−0.011 (2)	0.0357 (18)	0.0015 (19)
C12	0.101 (3)	0.065 (2)	0.0548 (17)	0.008 (2)	0.0177 (19)	0.0020 (19)
C13	0.0633 (18)	0.071 (2)	0.0594 (17)	0.0094 (19)	0.0132 (15)	−0.0004 (18)
C14	0.0546 (15)	0.0540 (19)	0.0515 (14)	−0.0038 (16)	0.0195 (13)	−0.0051 (15)

### (I) 1-[(1R)-2-Hydroxy-1-phenylethyl]piperidin-4-one Geometric parameters (Å, º)

**Table:**

N1—C2	1.466 (3)	C6—H6B	0.9700
N1—C6	1.469 (3)	C7—C9	1.514 (4)
N1—C7	1.481 (3)	C7—C8	1.525 (4)
O1—C4	1.217 (4)	C7—H7A	0.9800
O2—C8	1.416 (3)	C8—H8A	0.9700
O2—H2	0.9051	C8—H8B	0.9700
C2—C3	1.530 (4)	C9—C10	1.387 (4)
C2—H2B	0.9700	C9—C14	1.398 (3)
C2—H2C	0.9700	C10—C11	1.392 (5)
C3—C4	1.487 (5)	C10—H10A	0.9300
C3—H3A	0.9700	C11—C12	1.376 (4)
C3—H3B	0.9700	C11—H11A	0.9300
C4—C5	1.483 (5)	C12—C13	1.373 (5)
C5—C6	1.524 (3)	C12—H12A	0.9300
C5—H5A	0.9700	C13—C14	1.385 (4)
C5—H5B	0.9700	C13—H13A	0.9300
C6—H6A	0.9700	C14—H14A	0.9300
			
C2—N1—C6	109.7 (2)	N1—C7—C9	116.1 (2)
C2—N1—C7	111.57 (19)	N1—C7—C8	108.1 (2)
C6—N1—C7	113.85 (17)	C9—C7—C8	114.2 (2)
C8—O2—H2	104.7	N1—C7—H7A	105.9
N1—C2—C3	110.9 (2)	C9—C7—H7A	105.9
N1—C2—H2B	109.5	C8—C7—H7A	105.9
C3—C2—H2B	109.5	O2—C8—C7	111.4 (2)
N1—C2—H2C	109.5	O2—C8—H8A	109.4
C3—C2—H2C	109.5	C7—C8—H8A	109.4
H2B—C2—H2C	108.0	O2—C8—H8B	109.4
C4—C3—C2	111.2 (2)	C7—C8—H8B	109.4
C4—C3—H3A	109.4	H8A—C8—H8B	108.0
C2—C3—H3A	109.4	C10—C9—C14	117.2 (2)
C4—C3—H3B	109.4	C10—C9—C7	120.1 (2)
C2—C3—H3B	109.4	C14—C9—C7	122.7 (2)
H3A—C3—H3B	108.0	C9—C10—C11	121.4 (3)
O1—C4—C5	122.6 (3)	C9—C10—H10A	119.3
O1—C4—C3	122.3 (4)	C11—C10—H10A	119.3
C5—C4—C3	115.1 (3)	C12—C11—C10	120.2 (3)
C4—C5—C6	111.1 (2)	C12—C11—H11A	119.9
C4—C5—H5A	109.4	C10—C11—H11A	119.9
C6—C5—H5A	109.4	C13—C12—C11	119.5 (3)
C4—C5—H5B	109.4	C13—C12—H12A	120.2
C6—C5—H5B	109.4	C11—C12—H12A	120.2
H5A—C5—H5B	108.0	C12—C13—C14	120.4 (3)
N1—C6—C5	110.09 (19)	C12—C13—H13A	119.8
N1—C6—H6A	109.6	C14—C13—H13A	119.8
C5—C6—H6A	109.6	C13—C14—C9	121.4 (3)
N1—C6—H6B	109.6	C13—C14—H14A	119.3
C5—C6—H6B	109.6	C9—C14—H14A	119.3
H6A—C6—H6B	108.2		
			
C6—N1—C2—C3	61.7 (3)	N1—C7—C8—O2	−50.9 (3)
C7—N1—C2—C3	−171.2 (2)	C9—C7—C8—O2	178.2 (2)
N1—C2—C3—C4	−52.6 (4)	N1—C7—C9—C10	91.9 (3)
C2—C3—C4—O1	−131.9 (3)	C8—C7—C9—C10	−141.3 (2)
C2—C3—C4—C5	46.5 (4)	N1—C7—C9—C14	−86.8 (3)
O1—C4—C5—C6	130.5 (3)	C8—C7—C9—C14	40.1 (3)
C3—C4—C5—C6	−47.9 (4)	C14—C9—C10—C11	0.1 (4)
C2—N1—C6—C5	−62.8 (3)	C7—C9—C10—C11	−178.6 (3)
C7—N1—C6—C5	171.4 (2)	C9—C10—C11—C12	−1.1 (5)
C4—C5—C6—N1	55.1 (4)	C10—C11—C12—C13	1.0 (5)
C2—N1—C7—C9	−73.2 (3)	C11—C12—C13—C14	0.0 (5)
C6—N1—C7—C9	51.5 (3)	C12—C13—C14—C9	−1.0 (5)
C2—N1—C7—C8	157.0 (2)	C10—C9—C14—C13	1.0 (4)
C6—N1—C7—C8	−78.3 (3)	C7—C9—C14—C13	179.6 (3)

### (I) 1-[(1R)-2-Hydroxy-1-phenylethyl]piperidin-4-one Hydrogen-bond geometry (Å, º)

**Table:**

*D*—H···*A*	*D*—H	H···*A*	*D*···*A*	*D*—H···*A*
O2—H2···N1	0.91	2.22	2.764 (3)	118
C2—H2*B*···O2^i^	0.97	2.65	3.460 (4)	142
C3—H3*A*···O1^ii^	0.97	2.49	3.246 (4)	135

Symmetry codes: (i) *x*, *y*+1, *z*; (ii) −*x*+2, *y*−1/2, −*z*+1.

### (II) 8-[(1S)-1-Phenylethyl]-1,4-dioxa-8-azaspiro[4.5]decane-7-thione Crystal data

**Table:**

C_15_H_19_NO_2_S	*D*_x_ = 1.279 Mg m^−^^3^
*M**_r_* = 277.37	Mo *K*α radiation, λ = 0.71073 Å
Orthorhombic, *P*2_1_2_1_2_1_	Cell parameters from 58 reflections
*a* = 5.9731 (13) Å	θ = 4.7–12.5°
*b* = 14.948 (3) Å	µ = 0.22 mm^−^^1^
*c* = 16.127 (3) Å	*T* = 296 K
*V* = 1439.9 (5) Å^3^	Irregular, colourless
*Z* = 4	0.60 × 0.38 × 0.36 mm
*F*(000) = 592	

### (II) 8-[(1S)-1-Phenylethyl]-1,4-dioxa-8-azaspiro[4.5]decane-7-thione Data collection

**Table:**

Bruker P4 diffractometer	2007 reflections with *I* > 2σ(*I*)
Radiation source: fine-focus sealed tube	*R*_int_ = 0.029
Graphite monochromator	θ_max_ = 27.5°, θ_min_ = 1.9°
2θ/ω scans	*h* = −7→3
Absorption correction: ψ scan (*XSCANS*; Fait, 1996)	*k* = −19→19
*T*_min_ = 0.760, *T*_max_ = 0.922	*l* = −20→20
3886 measured reflections	3 standard reflections every 97 reflections
2631 independent reflections	intensity decay: 1.5%

### (II) 8-[(1S)-1-Phenylethyl]-1,4-dioxa-8-azaspiro[4.5]decane-7-thione Refinement

**Table:**

Refinement on *F*^2^	Hydrogen site location: inferred from neighbouring sites
Least-squares matrix: full	H-atom parameters constrained
*R*[*F*^2^ > 2σ(*F*^2^)] = 0.044	*w* = 1/[σ^2^(*F*_o_^2^) + (0.0668*P*)^2^ + 0.0631*P*] where *P* = (*F*_o_^2^ + 2*F*_c_^2^)/3
*wR*(*F*^2^) = 0.120	(Δ/σ)_max_ < 0.001
*S* = 1.06	Δρ_max_ = 0.19 e Å^−^^3^
2631 reflections	Δρ_min_ = −0.24 e Å^−^^3^
174 parameters	Extinction correction: *SHELXL2014* (Sheldrick, 2015), Fc^*^=kFc[1+0.001xFc^2^λ^3^/sin(2θ)]^-1/4^
0 restraints	Extinction coefficient: 0.014 (4)
0 constraints	Absolute structure: Flack *x* determined using 483 quotients [(*I*^+^)-(*I*^-^)]/[(*I*^+^)+(*I*^-^)] (Parsons *et al.*, 2013)
Primary atom site location: structure-invariant direct methods	Absolute structure parameter: 0.08 (7)
Secondary atom site location: difference Fourier map	

### (II) 8-[(1S)-1-Phenylethyl]-1,4-dioxa-8-azaspiro[4.5]decane-7-thione Fractional atomic coordinates and isotropic or equivalent isotropic displacement parameters (Å^2^)

**Table:**

	*x*	*y*	*z*	*U*_iso_*/*U*_eq_	
S1	0.90206 (18)	0.38336 (6)	0.64284 (6)	0.0676 (3)	
N1	0.5705 (4)	0.49810 (13)	0.61607 (13)	0.0409 (6)	
C2	0.7114 (5)	0.46066 (19)	0.66903 (18)	0.0435 (7)	
C3	0.6996 (6)	0.4875 (2)	0.75975 (18)	0.0522 (8)	
H3A	0.6350	0.4383	0.7909	0.063*	
H3B	0.8509	0.4964	0.7800	0.063*	
C4	0.5660 (6)	0.57054 (18)	0.77730 (17)	0.0459 (7)	
C5	0.3483 (6)	0.5637 (2)	0.73219 (19)	0.0532 (8)	
H5A	0.2547	0.6147	0.7456	0.064*	
H5B	0.2702	0.5098	0.7490	0.064*	
C6	0.3927 (6)	0.5612 (2)	0.63970 (17)	0.0564 (8)	
H6A	0.2557	0.5447	0.6113	0.068*	
H6B	0.4342	0.6207	0.6213	0.068*	
C7	0.5849 (6)	0.47717 (17)	0.52588 (16)	0.0454 (7)	
H7A	0.7363	0.4545	0.5156	0.054*	
C8	0.4240 (8)	0.40247 (19)	0.5035 (2)	0.0663 (10)	
H8A	0.4611	0.3498	0.5346	0.099*	
H8B	0.4353	0.3898	0.4453	0.099*	
H8C	0.2738	0.4205	0.5164	0.099*	
C9	0.5595 (6)	0.56255 (18)	0.47498 (16)	0.0434 (7)	
C10	0.3737 (6)	0.5799 (2)	0.42688 (18)	0.0525 (8)	
H10A	0.2566	0.5390	0.4250	0.063*	
C11	0.3612 (8)	0.6593 (2)	0.38083 (18)	0.0635 (10)	
H11A	0.2348	0.6712	0.3490	0.076*	
C12	0.5336 (8)	0.7192 (2)	0.3824 (2)	0.0689 (11)	
H12A	0.5256	0.7714	0.3512	0.083*	
C13	0.7186 (8)	0.7023 (2)	0.4302 (2)	0.0684 (11)	
H13A	0.8356	0.7433	0.4319	0.082*	
C14	0.7310 (6)	0.6238 (2)	0.4760 (2)	0.0559 (8)	
H14A	0.8576	0.6125	0.5079	0.067*	
O15	0.6883 (5)	0.64740 (15)	0.75095 (14)	0.0743 (8)	
C16	0.6637 (12)	0.7128 (3)	0.8120 (3)	0.120 (2)	
H16A	0.5799	0.7632	0.7902	0.144*	
H16B	0.8095	0.7341	0.8297	0.144*	
C17	0.5447 (8)	0.6734 (2)	0.8821 (2)	0.0666 (11)	
H17A	0.6241	0.6845	0.9335	0.080*	
H17B	0.3948	0.6979	0.8865	0.080*	
O18	0.5371 (4)	0.57972 (13)	0.86428 (12)	0.0553 (6)	

### (II) 8-[(1S)-1-Phenylethyl]-1,4-dioxa-8-azaspiro[4.5]decane-7-thione Atomic displacement parameters (Å^2^)

**Table:**

	*U*^11^	*U*^22^	*U*^33^	*U*^12^	*U*^13^	*U*^23^
S1	0.0725 (6)	0.0688 (6)	0.0615 (5)	0.0339 (5)	−0.0020 (5)	−0.0005 (4)
N1	0.0465 (14)	0.0369 (11)	0.0393 (11)	0.0038 (12)	0.0012 (12)	0.0018 (10)
C2	0.0438 (16)	0.0412 (14)	0.0456 (15)	0.0002 (14)	−0.0005 (14)	0.0061 (12)
C3	0.0563 (19)	0.0588 (18)	0.0414 (15)	0.0078 (18)	−0.0023 (15)	0.0039 (14)
C4	0.0578 (19)	0.0404 (14)	0.0393 (13)	−0.0059 (16)	0.0102 (15)	0.0043 (12)
C5	0.054 (2)	0.0551 (17)	0.0501 (16)	0.0096 (17)	0.0064 (16)	0.0031 (14)
C6	0.065 (2)	0.0605 (18)	0.0440 (15)	0.0239 (18)	0.0021 (18)	0.0016 (14)
C7	0.0583 (19)	0.0392 (13)	0.0386 (13)	0.0007 (16)	0.0001 (16)	0.0003 (11)
C8	0.093 (3)	0.0462 (17)	0.0593 (18)	−0.013 (2)	−0.008 (2)	0.0002 (13)
C9	0.0552 (19)	0.0385 (13)	0.0365 (12)	0.0012 (15)	0.0025 (14)	−0.0025 (11)
C10	0.059 (2)	0.0548 (16)	0.0431 (14)	−0.0025 (17)	−0.0062 (17)	0.0039 (13)
C11	0.074 (3)	0.071 (2)	0.0461 (16)	0.010 (2)	−0.0115 (18)	0.0122 (16)
C12	0.099 (3)	0.0540 (18)	0.0543 (17)	0.003 (2)	0.008 (2)	0.0160 (16)
C13	0.084 (3)	0.0496 (19)	0.072 (2)	−0.015 (2)	0.003 (2)	0.0105 (16)
C14	0.0551 (19)	0.0547 (18)	0.0580 (18)	−0.0086 (18)	−0.0075 (17)	0.0077 (16)
O15	0.108 (2)	0.0544 (12)	0.0601 (13)	−0.0289 (14)	0.0275 (15)	−0.0022 (11)
C16	0.200 (7)	0.062 (2)	0.097 (3)	−0.060 (4)	0.057 (4)	−0.022 (2)
C17	0.100 (3)	0.0484 (17)	0.0516 (17)	−0.005 (2)	0.006 (2)	−0.0045 (14)
O18	0.0793 (17)	0.0454 (10)	0.0412 (10)	−0.0079 (12)	0.0118 (11)	0.0009 (8)

### (II) 8-[(1S)-1-Phenylethyl]-1,4-dioxa-8-azaspiro[4.5]decane-7-thione Geometric parameters (Å, º)

**Table:**

S1—C2	1.677 (3)	C8—H8B	0.9600
N1—C2	1.323 (4)	C8—H8C	0.9600
N1—C6	1.471 (4)	C9—C14	1.375 (5)
N1—C7	1.490 (3)	C9—C10	1.379 (5)
C2—C3	1.519 (4)	C10—C11	1.402 (4)
C3—C4	1.503 (4)	C10—H10A	0.9300
C3—H3A	0.9700	C11—C12	1.365 (6)
C3—H3B	0.9700	C11—H11A	0.9300
C4—O18	1.420 (3)	C12—C13	1.370 (6)
C4—O15	1.426 (3)	C12—H12A	0.9300
C4—C5	1.493 (5)	C13—C14	1.388 (5)
C5—C6	1.515 (4)	C13—H13A	0.9300
C5—H5A	0.9700	C14—H14A	0.9300
C5—H5B	0.9700	O15—C16	1.395 (4)
C6—H6A	0.9700	C16—C17	1.459 (5)
C6—H6B	0.9700	C16—H16A	0.9700
C7—C8	1.517 (4)	C16—H16B	0.9700
C7—C9	1.525 (4)	C17—O18	1.430 (3)
C7—H7A	0.9800	C17—H17A	0.9700
C8—H8A	0.9600	C17—H17B	0.9700
			
C2—N1—C6	124.3 (2)	C7—C8—H8B	109.5
C2—N1—C7	120.3 (3)	H8A—C8—H8B	109.5
C6—N1—C7	115.4 (2)	C7—C8—H8C	109.5
N1—C2—C3	118.7 (3)	H8A—C8—H8C	109.5
N1—C2—S1	124.1 (2)	H8B—C8—H8C	109.5
C3—C2—S1	117.1 (2)	C14—C9—C10	118.8 (3)
C4—C3—C2	115.1 (2)	C14—C9—C7	118.5 (3)
C4—C3—H3A	108.5	C10—C9—C7	122.7 (3)
C2—C3—H3A	108.5	C9—C10—C11	120.0 (3)
C4—C3—H3B	108.5	C9—C10—H10A	120.0
C2—C3—H3B	108.5	C11—C10—H10A	120.0
H3A—C3—H3B	107.5	C12—C11—C10	120.3 (4)
O18—C4—O15	106.2 (2)	C12—C11—H11A	119.8
O18—C4—C5	112.5 (3)	C10—C11—H11A	119.8
O15—C4—C5	110.8 (3)	C11—C12—C13	119.9 (3)
O18—C4—C3	109.3 (2)	C11—C12—H12A	120.0
O15—C4—C3	109.7 (3)	C13—C12—H12A	120.0
C5—C4—C3	108.3 (3)	C12—C13—C14	119.8 (4)
C4—C5—C6	109.2 (3)	C12—C13—H13A	120.1
C4—C5—H5A	109.8	C14—C13—H13A	120.1
C6—C5—H5A	109.8	C9—C14—C13	121.1 (3)
C4—C5—H5B	109.8	C9—C14—H14A	119.4
C6—C5—H5B	109.8	C13—C14—H14A	119.4
H5A—C5—H5B	108.3	C16—O15—C4	107.5 (3)
N1—C6—C5	113.4 (2)	O15—C16—C17	108.3 (3)
N1—C6—H6A	108.9	O15—C16—H16A	110.0
C5—C6—H6A	108.9	C17—C16—H16A	110.0
N1—C6—H6B	108.9	O15—C16—H16B	110.0
C5—C6—H6B	108.9	C17—C16—H16B	110.0
H6A—C6—H6B	107.7	H16A—C16—H16B	108.4
N1—C7—C8	110.5 (3)	O18—C17—C16	104.8 (3)
N1—C7—C9	110.1 (2)	O18—C17—H17A	110.8
C8—C7—C9	115.2 (3)	C16—C17—H17A	110.8
N1—C7—H7A	106.9	O18—C17—H17B	110.8
C8—C7—H7A	106.9	C16—C17—H17B	110.8
C9—C7—H7A	106.9	H17A—C17—H17B	108.9
C7—C8—H8A	109.5	C4—O18—C17	106.8 (2)
			
C6—N1—C2—C3	−3.5 (4)	C8—C7—C9—C14	−163.6 (3)
C7—N1—C2—C3	176.7 (3)	N1—C7—C9—C10	−110.5 (3)
C6—N1—C2—S1	175.5 (2)	C8—C7—C9—C10	15.2 (4)
C7—N1—C2—S1	−4.3 (4)	C14—C9—C10—C11	−0.6 (5)
N1—C2—C3—C4	−14.3 (4)	C7—C9—C10—C11	−179.4 (3)
S1—C2—C3—C4	166.7 (2)	C9—C10—C11—C12	0.9 (5)
C2—C3—C4—O18	170.4 (3)	C10—C11—C12—C13	−0.9 (6)
C2—C3—C4—O15	−73.6 (4)	C11—C12—C13—C14	0.8 (6)
C2—C3—C4—C5	47.6 (4)	C10—C9—C14—C13	0.5 (5)
O18—C4—C5—C6	175.7 (2)	C7—C9—C14—C13	179.3 (3)
O15—C4—C5—C6	57.1 (3)	C12—C13—C14—C9	−0.5 (5)
C3—C4—C5—C6	−63.4 (3)	O18—C4—O15—C16	−19.9 (5)
C2—N1—C6—C5	−13.5 (4)	C5—C4—O15—C16	102.5 (4)
C7—N1—C6—C5	166.3 (3)	C3—C4—O15—C16	−137.9 (4)
C4—C5—C6—N1	47.2 (4)	C4—O15—C16—C17	6.2 (6)
C2—N1—C7—C8	94.8 (4)	O15—C16—C17—O18	9.6 (6)
C6—N1—C7—C8	−85.0 (3)	O15—C4—O18—C17	26.1 (4)
C2—N1—C7—C9	−136.8 (3)	C5—C4—O18—C17	−95.3 (3)
C6—N1—C7—C9	43.4 (4)	C3—C4—O18—C17	144.4 (3)
N1—C7—C9—C14	70.7 (4)	C16—C17—O18—C4	−21.9 (5)

### (II) 8-[(1S)-1-Phenylethyl]-1,4-dioxa-8-azaspiro[4.5]decane-7-thione Hydrogen-bond geometry (Å, º)

**Table:**

*D*—H···*A*	*D*—H	H···*A*	*D*···*A*	*D*—H···*A*
C7—H7*A*···S1	0.98	2.51	3.019 (3)	112
C16—H16*B*···S1^i^	0.97	2.85	3.709 (5)	148

Symmetry code: (i) −*x*+2, *y*+1/2, −*z*+3/2.

## References

[bb1] Bolla, G., Mittapalli, S. & Nangia, A. (2014). *CrystEngComm*, **16**, 24–27.

[bb2] Busschaert, N., Wenzel, M., Light, M. E., Iglesias-Hernández, P., Pérez-Tomás, R. & Gale, P. A. (2011). *J. Am. Chem. Soc.* **133**, 14136–14148. 10.1021/ja205884y PMC3436094 21846096

[bb3] Chumakov, Yu. M., Samus’, N. M., Bocelli, G., Suponitskii, K. Yu., Tsapkov, V. I. & Gulya, A. P. (2006). *Russ. J. Coord. Chem.* **32**, 14–20.

[bb4] Das, U., Sakagami, H., Chu, Q., Wang, Q., Kawase, M., Selvakumar, P., Sharma, R. K. & Dimmock, J. R. (2010). *Bioorg. Med. Chem. Lett.* **20**, 912–917. 10.1016/j.bmcl.2009.12.076 PMC3276597 20064715

[bb5] Davis, F. A., Chao, B. & Rao, A. (2001). *Org. Lett.* **3**, 3169–3171. 10.1021/ol0164839 11574022

[bb6] Fait, J. (1996). *XSCANS*. Siemens Analytical X-ray Instruments Inc., Madison, Wisconsin, USA.

[bb7] Groom, C. R. & Allen, F. H. (2014). *Angew. Chem. Int. Ed.* **53**, 662–671. 10.1002/anie.201306438 24382699

[bb8] Gupta, P. K., Manral, L., Ganesan, K., Malhotra, R. C. & Sekhar, K. (2010). Patent WO 2009116084 A2.

[bb9] Krebs, B. & Gattow, G. (1965). *Z. Anorg. Allg. Chem.* **340**, 294–311.

[bb10] Kuze, N., Funahashi, H., Ogawa, M., Tajiri, H., Ohta, Y., Usami, T., Sakaizumi, T. & Ohashi, O. (1999). *J. Mol. Spectrosc.* **198**, 381–386. 10.1006/jmsp.1999.7959 10547320

[bb11] Parsons, S., Flack, H. D. & Wagner, T. (2013). *Acta Cryst.* B**69**, 249–259. 10.1107/S2052519213010014 PMC3661305 23719469

[bb12] Rajesh, K., Reddy, B. P. & Vijayakumar, V. (2012). *Ultrason. Sonochem* **19**, 522–531. 10.1016/j.ultsonch.2011.10.018 22129974

[bb13] Rajesh, K., Vijayakumar, V., Sarveswari, S., Narasimhamurthy, T. & Tiekink, E. R. T. (2010*a*). *Acta Cryst.* E**66**, o1306–o1307. 10.1107/S1600536810016570 PMC2979355 21579402

[bb14] Rajesh, K., Vijayakumar, V., Sarveswari, S., Narasimhamurthy, T. & Tiekink, E. R. T. (2010*b*). *Acta Cryst.* E**66**, o1988. 10.1107/S1600536810026681 PMC3007266 21588303

[bb15] Romero, N., Gnecco, D., Terán, J. & Bernès, S. (2013). *Acta Cryst.* E**69**, o408–o409. 10.1107/S1600536813004017 PMC3588428 23476588

[bb16] Romero, N., Gnecco, D., Terán, J., Juárez, J. & Galindo, A. (2007). *J. Sulfur Chem.* **28**, 239–243.

[bb17] Sanfilippo, P. J., McNally, J. J., Press, J. B., Fitzpatrick, L. J., Urbanski, M. J., Katz, L. B., Giardino, E., Falotico, R., Salata, J., Moore, J. B. Jr & Miller, W. (1992). *J. Med. Chem* **35**, 4425–4433. 10.1021/jm00101a020 1447742

[bb18] Shahani, T., Fun, H.-K., Ragavan, R. V., Vijayakumar, V. & Venkatesh, M. (2010). *Acta Cryst.* E**66**, o3233–o3234. 10.1107/S1600536810047215 PMC3011612 21589523

[bb19] Sheldrick, G. M. (2008). *Acta Cryst.* A**64**, 112–122. 10.1107/S0108767307043930 18156677

[bb20] Sheldrick, G. M. (2015). *Acta Cryst.* C**71**, 3–8.

[bb21] Thirumaran, S., Ramalingam, K., Bocelli, G. & Righi, L. (2009). *Polyhedron*, **28**, 263–268.

[bb22] Tortolani, D. R. & Poss, M. A. (1999). *Org. Lett.* **1**, 1261–1262.

[bb23] Vijayakumar, V., Rajesh, K., Suresh, J., Narasimhamurthy, T. & Lakshman, P. L. N. (2010). *Acta Cryst.* E**66**, o170. 10.1107/S1600536809052908 PMC2980215 21580057

[bb24] Weber, G. (1984). *J. Inclusion Phenom.* **1**, 339–347.

[bb25] Woydt, M., Rademacher, P., Brett, W. A. & Boese, R. (1991). *Acta Cryst.* C**47**, 1936–1938.

